# Developing a Competency List for General Practitioners/Family Physicians Providing Care for Dual‐Location Residents: Expert Consensus via the Delphi Technique

**DOI:** 10.1002/jgf2.70149

**Published:** 2026-07-07

**Authors:** Hirohisa Fujikawa, Junki Mizumoto, Hirotake Mori, Yuji Nishizaki, Yuichiro Yano, Toshio Naito

**Affiliations:** ^1^ Department of General Medicine Juntendo University Faculty of Medicine Bunkyo‐ku Japan; ^2^ Center for General Medicine Education, School of Medicine Keio University Shinjuku‐ku Japan; ^3^ Department of Medical Education Studies, International Research Center for Medical Education, Graduate School of Medicine The University of Tokyo Bunkyo‐ku Japan; ^4^ Department of Family Practice Ehime Seikyo Hospital Matsuyama Japan; ^5^ Division of Medical Education Juntendo University Faculty of Medicine Bunkyo‐ku Japan

**Keywords:** comprehensive care, continuity of care, dual‐location living, fragmented care, tolerance for ambiguity

## Abstract

**Background:**

Dual‐location living, in which individuals reside across two geographically distinct regions, is becoming increasingly prevalent in Japan. Consequently, general practitioners and family physicians (GPs/FPs) are more likely to encounter such patients, particularly in clinical settings located in resort areas and other non‐metropolitan communities. Nevertheless, competency frameworks specifically tailored to the care of dual‐location residents remain to be established.

**Methods:**

A Delphi method was employed to develop a competency list for GPs/FPs involved in dual‐location resident care. An expert panel of healthcare professionals with relevant clinical or educational experience in providing care for dual‐location residents in Nagano Prefecture was invited to participate in the online Delphi rounds. In Round 1, the panelists responded to an open‐ended questionnaire regarding competencies required for high‐quality dual‐location resident care. Responses were analyzed using qualitative content analysis to generate an initial competency list. In Round 2, the list was refined and confirmed by consensus.

**Results:**

A total of 36 participants completed Round 1, of whom 35 also completed Round 2. Through the Delphi process, a competency list comprising 10 items was developed. These competencies were organized into four domains, summarized using the acronym ROAM: Recognition of dual‐location context, Orchestration of dual‐location care, Alignment of care plan, and Multicultural professionalism.

**Conclusions:**

This study developed a consensus‐based competency framework for GPs/FPs involved in dual‐location resident care. The ROAM model may serve as a helpful reference for clinical practice, medical education, and future research, while further studies are warranted to examine its applicability across different settings.

## 1 Introduction

In recent years, mobility across multiple living locations has attracted growing attention internationally. Particularly, the term “seasonal migrants” or “snowbirds” is used to describe older adults who engage in seasonal mobility [1]. A body of research has previously described the prevalence of seasonal migration, patterns of healthcare utilization, and the challenges associated with accessing care across locations [2, 3, 4, 5]. For instance, approximately 3% of older adults in Ontario migrate to warmer destinations during winter, and seasonal migrants have been reported to utilize healthcare services across multiple settings [5]. These findings suggest that geographically mobile lifestyles represent an increasingly relevant issue for healthcare systems in several countries.

A similar phenomenon has been observed in Japan, where living in two places, commonly termed dual‐location living or dual habitation, has been of great interest in recent years [6, 7]. Dual‐location living refers to a lifestyle of living in both urban and rural areas (typically an urban residence and a second base in a rural area) [6]. Many individuals initiated a move from urban to rural areas in the wake of the 2008 Lehman Shock and 2011 Great East Japan Earthquake [8]. Interest subsequently accelerated in response to the social and workstyle changes brought on by the coronavirus disease 2019 pandemic, including the normalization of remote work and changes in lifestyle and sense of values [7, 9]. Additionally, both public and private initiatives have sought to promote dual‐location living [7] the Ministry of Land, Infrastructure, Transport, and Tourism has issued national policy guidance to promote dual‐location living [10]; several prefectures have introduced measures to promote it [11, 12]; and in 2024 the National Public‐Private Partnership Platform for Promoting Dual Habitation was established to facilitate collaboration and information sharing related to dual‐location living [13]. The number of individuals who reside across two regions and utilize healthcare services in both locations is thus expected to continue growing.

As a consequence, general practitioners and family physicians (GPs/FPs) are increasingly likely to encounter dual‐location residents, particularly in clinical settings located in resort areas and other non‐metropolitan communities. Although dual‐location living encompasses diverse mobility patterns, the present study primarily focuses on patients who receive healthcare across two living locations, often in association with seasonal movement between urban and rural areas. For example, Nagano prefecture, which contains numerous resort and second‐home areas, represents a typical setting in which dual habitation is relatively common. In such regions, the encounters span a broad clinical spectrum, ranging from routine follow‐up visits to, importantly, unscheduled acute visits. In many of these settings, GPs/FPs function as the first point of contact and need to address a wide range of health concerns. They are therefore expected to play a crucial role in providing care for dual‐location residents. For clarity, the term “GPs/FPs” is used in this study to refer to physicians who provide comprehensive care, taking a multisystemic and holistic approach to patients with consideration to their lifestyle and broader social and/or family context, rather than being limited to the silo of a particular medical specialty [14, 15].

However, care for dual‐location residents raises various concerns about potentially adverse clinical outcomes. For instance, dual habitants are likely to receive care from multiple physicians, raising concerns about fragmented care. Fragmented care refers to the lack of sufficient coordination among healthcare providers in single‐patient care [16]. This can lead to inefficient and ineffective care, such as an increase in duplicated tests, emergency department visits, medical costs, and hospitalizations [17, 18, 19]. Although information continuity is a vital component of the continuity of care [20], it can be compromised in dual‐location care. Information discontinuity is particularly evident when referral letters or medication lists are unavailable during encounters. Despite these challenges, GPs/FPs are required to deliver safe and high‐quality care for dual‐location residents.

A number of competency frameworks for GPs/FPs have been developed. For example, the American Board of Family Medicine outlines core competency domains that family physicians are expected to demonstrate across comprehensive and continuity care [21]. The Royal Australian College of General Practitioners defines GP competence across five domains and a set of high‐level core skills, with assessment structured around 10 core clinical competencies (e.g., communication, clinical reasoning, professionalism), along with contextual units focused on Indigenous and rural health [22]. In Japan, the Japan Primary Care Association, a recognized certifying body for primary care physicians, and the Japanese Medical Specialty Board, a centralized body that standardizes the training and certification of medical specialists in Japan, list the following seven qualities and abilities that should be developed during GP/FP residency training: a comprehensive and integrated approach; clinical competence in addressing common health problems; person‐centered medicine/care; a collaboration‐focused approach; a community‐oriented approach, including community‐based integrated care; professional standards that serve the public interest; and the ability to adapt to diverse clinical settings [23, 24]. These frameworks offer an important foundation for understanding what competencies GPs/FPs should develop.

Despite the relevance of these frameworks, they are unlikely to adequately reflect the demands associated with dual‐location living resident care. Furthermore, while prior studies on seasonal migrants and snowbirds have described patterns of healthcare utilization and challenges associated with receiving care across locations, little attention has been paid to the competencies required of GPs/FPs caring for these geographically mobile patients. These include the need to address clinical and logistical needs across geographically fragmented care environments and practical constraints of incomplete information. Accordingly, the need to establish a competency framework specifically tailored to GPs/FPs providing care for dual habitants in Japan is urgent.

Here, we aimed to develop a competency list for GPs/FPs involved in the care of dual‐location residents in Japan. In developing this list, we sought to provide a practical reference for (1) clinical practice improvement in resort areas and other relevant settings, (2) medical education for GPs/FPs who increasingly encounter dual‐location residents, and (3) future research initiatives on care quality for this population.

## 2 Methods

### 2.1 Study Context

For this study, we recruited GPs/FPs and other healthcare professionals who had prior experience in general practice/family medicine care in Karuizawa Town, Chino City, and Hara Village, Nagano Prefecture, Japan. The selection of these settings was based on the following factors:

Nagano Prefecture is located in the Chubu region, which is situated in the central part of Japan's main island. The prefecture is characterized by its natural richness and highest average altitude among all 47 prefectures in Japan. It has a cooler climate than coastal areas and most of the large cities and contains several resort areas that attract seasonal residents and second‐home owners from urban areas. Karuizawa Town has historically flourished as a summer resort since the Meiji era; the opening of a Shinkansen station brought a notable increase in the number of immigrants from the Tokyo area [25], and the ride from Tokyo station to Karuizawa station now takes only approximately 1 h. Chino City includes the Tateshina Highlands, a well‐known resort and second‐home area, and nearby Hara Village also attracts visitors and seasonal residents [26]. Accordingly, healthcare institutions in these areas frequently encounter dual‐location residents who seek care during temporary stays.

### 2.2 Reflexivity and Research Paradigm

The first author (MD, PhD) is a GP/FP and researcher in medical education who has comprehensive experience in providing care for dual‐location residents in facilities in Karuizawa Town, Chino City, and Hara Village. This experience aided the conceptualization of the study and facilitated interpretation of participants' responses, particularly regarding the practical challenges encountered in dual‐location resident care. The second author (MD, PhD) is also a GP/FP and researcher in medical education. The third (MD, PhD), fourth (MD, MPH, PhD), fifth (MD, PhD), and sixth (MD, PhD) authors were all experienced GPs/FPs with a background in clinical practice, medical education, and research. While the second to sixth authors had no direct experience providing care for dual‐location residents in study settings, their perspectives allowed for triangulation with the first author, who was more closely connected to the local context and clinical realities of dual‐location resident care in Nagano Prefecture.

The present study was informed by a pragmatism paradigm, which emphasizes the generation of knowledge that is useful in addressing practical issues in real‐world settings [14, 27]. Because our objective was to develop a competency framework that could inform real‐world clinical practice, we combined qualitative exploration in Round 1 with consensus‐based quantitative evaluation in Round 2 (as detailed later).

### 2.3 The Delphi Method and Data Collection and Analyses

From March 3 to April 2, 2026, a Delphi process was implemented to reach a consensus on a competencies framework specifically tailored to GPs/FPs providing care for dual habitants [28]. The Delphi technique is a consensus‐building method that is widely employed in social sciences and health‐related studies [29]. The technique gathers a group of experts as panelists on a specific problem and systematically obtains consensus‐based opinions employing questionnaires. It is composed of repeated questionnaires (called rounds) and anonymous feedback summarized according to group responses. Because no previous studies had identified or described the competencies specifically required for GPs/FPs providing care for dual‐location residents, we considered it preferable to elicit competencies directly from experts rather than relying primarily on a researcher‐generated list. Therefore, we adopted a classical Delphi approach, in which the first round begins with an open‐ended question rather than a researcher‐generated list of candidate competencies [30]. This study followed the Guidance on Conducting and Reporting Delphi Studies [31].

In Round 1, in accordance with the classical Delphi approach, we did not present any predetermined competency items to the participants. Instead, participants were asked to freely describe competencies that they considered relevant for GPs/FPs who provide quality care for dual‐location residents (Data S1). This includes regular patient visits with referral letters from physicians at other locations, as well as the management of patients who present without an appointment or referral letter. The participants described their opinions on an open‐ended questionnaire. The first and second authors analyzed the responses to the questionnaire using qualitative content analysis with input from other authors. Discrepancies in coding and category development were discussed among the authors until agreement was reached. Based on the results of the content analysis, an initial competency list was developed for subsequent evaluation in Round 2.

In Round 2, participants were asked to rate items in the list in terms of their importance for quality care for dual‐location residents. Responses were recorded on five‐point Likert scales from 1 = absolutely unimportant to 5 = absolutely important. The participants were also asked to add free‐text comments as necessary (Data S2). Consistent with standard practice, rounds were held until a panelist consensus was reached [32]. The following predefined criteria were employed to determine whether or not a consensus had been obtained: (i) a mean score of 4 or more, (ii) a standard deviation of less than 1, and (iii) scores of 4 or 5 from 75% or more of the panelists [33, 34, 35]. Because all items met the predefined consensus criteria in Round 2, no additional rating round was conducted (as described below).

Throughout the Delphi process, we used SurveyMonkey as a web‐based survey instrument for both Rounds 1 and 2. Panelists were given approximately 1 week to complete each questionnaire. Non‐respondents were provided reminder emails a few days before each deadline. All questionnaires used in Rounds 1 and 2 were administered in Japanese. The competency items presented to the panelists during the Delphi rounds were therefore developed and evaluated in Japanese. The final competency list was subsequently translated into English by the researchers and professional translators for reporting purposes. Quantitative analysis was conducted using SPSS version 30.0.0.0 (IBM Corp), and qualitative content analysis was performed using Microsoft Excel for Mac version 16.101.3.

### 2.4 Panelists

A Delphi panel should consist of individuals with sufficient knowledge or skills related to the study's focus [36]. While no rule to define the number of panelists has been defined, a panel of 20–50 participants is most frequently recommended [37].

In the present study, purposive and snowball sampling were used to ensure sufficient diversity of panelists in terms of gender, age, and professional background. Eligibility criteria were predefined as prior clinical experience in providing care for dual‐location residents and/or experience in educating trainees involved in such care within GP/FP outpatient settings.

We first developed a list of potential panelists who met these criteria and invited them via email (purposive sampling). In the email, we asked them to participate in the study and provide the names of other potential panelists who met the same eligibility criteria (snowball sampling). To minimize the risk of including ineligible participants through snowball sampling, all nominated individuals were screened against the predefined eligibility criteria before inclusion, namely, prior clinical experience in providing care for dual‐location residents and/or experience in educating trainees involved in such care within GP/FP outpatient settings.

The final Delphi panel consisted primarily of physicians with experience in clinical practice and medical education, together with a small number of nurses and medical administrative staff. All participants were considered to possess sufficient knowledge and experience relevant to the study topic.

### 2.5 Ethical Considerations

Ethical clearance was obtained from the Ethics Committee of Juntendo University Faculty of Medicine (No. E25‐0037). All study participants provided written informed consent by ticking a consent box on the online questionnaire.

## 3 Results

We recruited 37 individuals for the panel, of whom 36 consented to participate. 21 were from purposive sampling and 15 from snowball sampling. Mean age (standard deviation) was 41.8 (11.9) years. Table 1 shows the characteristics of the panelists.

**TABLE 1:** Profile of the panelists.

Characteristics	Value
Gender, *n* (%)	
Man	23 (63.9)
Woman	13 (36.1)
Mean (standard deviation) age (years)	41.8 (11.9)
Age range (years)	26–72
Professions, *n* (%)	
Teaching physician	23 (63.9)
Senior medical resident	5 (13.9)
Junior medical resident	3 (8.3)
Nurse	4 (11.1)
Medical clerk	1 (2.8)

### 3.1 Round 1 of the Delphi Round

All 36 panelists completed the online questionnaire in Round 1. They provided a total of 236 comments on GP/FP quality of care for dual‐location residents. The comments were subsequently analyzed by qualitative content analysis to produce an initial competency list. This list was further developed into 10 items under the four categories of (i) recognition of dual‐location context, (ii) orchestration of dual‐location care, (iii) alignment of care plan, and (iv) multicultural professionalism.

### 3.2 Round 2 of the Delphi Round

In Round 2, 35 of 36 panelists (97.2%) provided responses to the questionnaire. In this round, we confirmed that all ten items met the predefined consensus criteria (Table 2), although some minor modifications were suggested. The list was revised in accordance with the suggestions. Instead of conducting Round 3, the revised list was distributed to the 35 Delphi panelists, all of whom approved the final version (Data S3).

**TABLE 2:** Results of the Delphi Round 2.

Item	Mean	Standard deviation	Respondents selecting 4 or 5, *N* (%)
1. The physician can comprehend the background, lifestyle patterns, and family/support systems of the patient	4.83	0.45	34 (97.1)
2. The physician can consider the impact of regional differences (e.g., climate and culture) on health^a^	4.34	0.84	31 (88.6)
3. The physician can collect necessary information from the patient and medical institutions at the other location	4.77	0.49	34 (97.1)
4. The physician can appropriately share information with medical institutions at the other location	4.86	0.43	34 (97.1)
5. The physician can adjust the division of roles and level of involvement in care between the two locations according to the situation	4.57	0.50	35 (100)
6. The physician can prioritize necessary interventions even when information is insufficient	4.43	0.88	31 (88.6)
7. The physician can coordinate with the patient regarding healthcare‐seeking behaviors and approaches to healthcare associated with a dual‐location lifestyle	4.34	0.84	29 (82.9)
8. The physician can discuss future treatment plans and daily life with the patient	4.37	0.69	31 (88.6)
9. The physician can adjust their own perceptions, given the social and cultural backgrounds of the patient associated with dual‐location living and differences in expectations regarding healthcare	4.11	0.99	27 (77.1)
10. The physician can provide care that takes into account the social and cultural backgrounds of the patient associated with dual‐location living and differences in expectations regarding healthcare	4.34	0.94	31 (88.6)

^a^ A minor modification was suggested for this item in this Delphi round. As a result, it was changed to “The physician can consider the impact of regional differences between the two locations on health and daily life”.

## 4 Discussion

**FIGURE 1:**
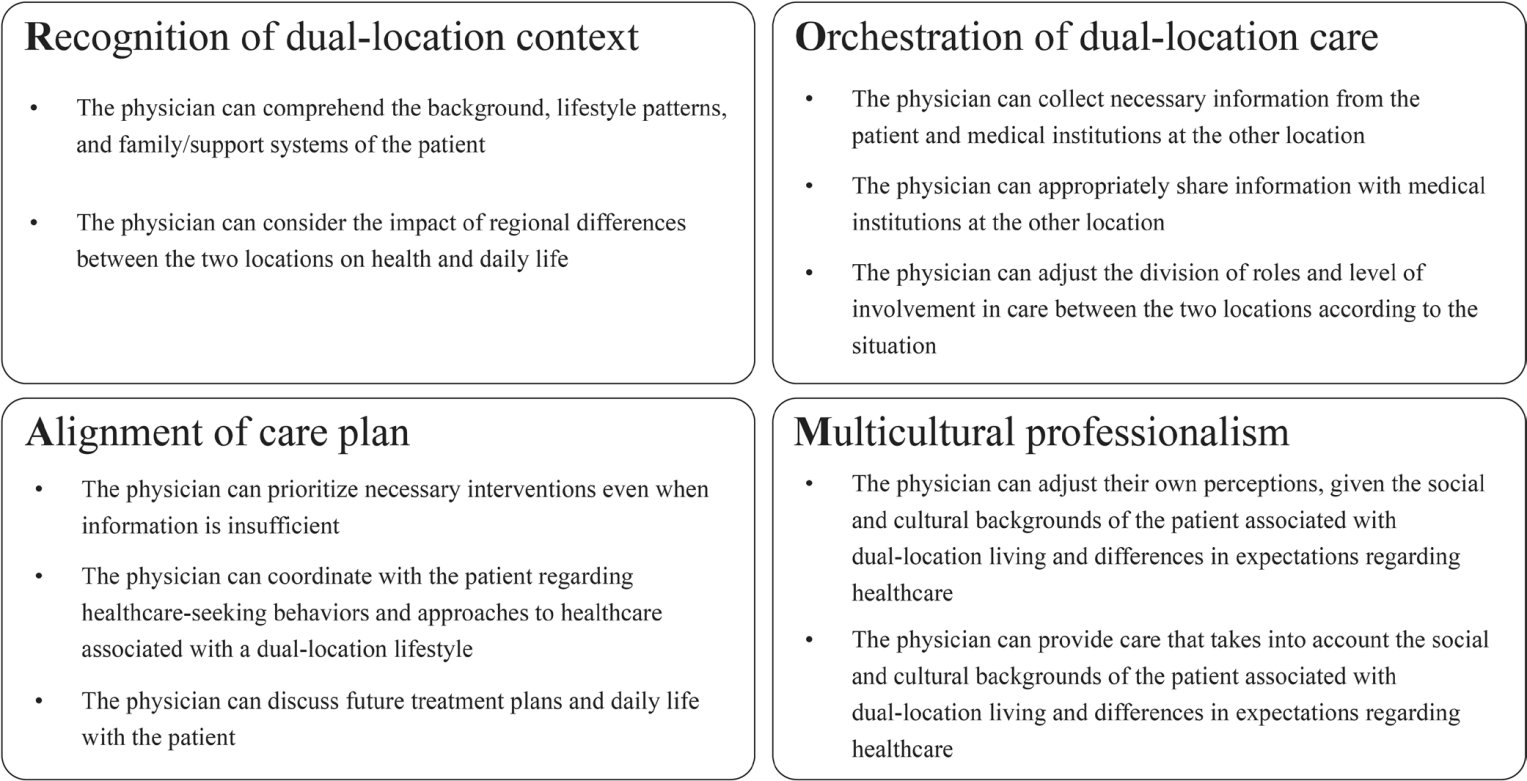
The ROAM model: recognition of dual‐location context; orchestration of dual‐location care; alignment of care plan; and multicultural professionalism.

This study aimed to identify a comprehensive and validated list of competencies for the provision of care for dual‐location residents in GP/FP outpatient settings. A competency list comprising 10 items was developed via the Delphi method. This list can be abbreviated using the acronym ROAM (Recommended cOmpetencies for cAre of Mobile patients): Recognition of dual‐location context; Orchestration of dual‐location care; Alignment of care plan; and Multicultural professionalism (Figure 1).

The domain “Recognition of dual‐location context” suggests the importance of comprehending the patient's life across multiple settings. In the GP/FP model, comprehensive and contextualized care has been emphasized as essential for addressing patients' needs [38]. For example, prior implementation research in Japan indicated that family medicine could improve the comprehensiveness of healthcare and address the issue of fragmentation of care [39]. Our study extends this existing evidence by explicitly addressing the need to recognize variability across contexts in the care of dual‐location residents. Such patients reside across two geographically distinct settings rather than a single location, and may experience differences in climate, culture, and healthcare accessibility between locations, all of which are likely to influence their health and healthcare‐seeking behaviors.

The “Orchestration of dual‐location care” domain highlights the necessity of managing care that may inherently be fragmented in dual‐location care settings. Care fragmentation poses a substantial challenge, particularly in this era of multimorbidity [40, 41]. Prior research has shown that care fragmentation arises from diverse interacting factors at the patient, provider, medical institution, and healthcare system levels, and that most of these are not directly related to medical need [42]. The findings suggest that care fragmentation is not simply a consequence of clinical complexity, but is shaped by broader contextual conditions. To date, however, research has mainly focused on care fragmentation within single geographic settings. The present study is of note because it extends prior evidence by shedding light on dual‐location living settings as a context in which fragmentation is not incidental but structurally likely to occur.

The domain of “Alignment of care plan” captures the competency of clinical decision‐making and negotiation of care in the context of dual‐location living, where clinical situations are not always well‐defined and may involve ambiguity. Tolerance for ambiguity has been identified as a crucial attribute of GPs/FPs, which allows them to practice effectively despite insufficient information and variable contexts [43]. In dual‐location resident care, GPs/FPs should prioritize necessary interventions, coordinate healthcare‐seeking behaviors across settings, and discuss future care and daily life with patients whose living contexts are fluid. Thus, alignment involves navigating ambiguous situations while continuously adjusting care plans in accordance with patients' changing contexts across locations.

The “Multicultural professionalism” domain reflects the necessity of recognizing and responding to differences in patients' social and cultural backgrounds, as well as variations in expectations towards healthcare. Cultural competence has been widely recognized as an important attribute of GP/FP [44, 45], particularly in addressing health disparities associated with social determinants of health [46]. In this context, prior discussions have primarily focused on challenges related to patients with social disadvantage. In dual‐location living, however, patients may span a wide range of social backgrounds, including those with relatively good economic and logistical resources, such as individuals maintaining second homes in resort areas. Such patients may bring different expectations to healthcare that do not always align with local practice environments. Our findings suggest that, under these conditions, multicultural professionalism requires GPs/FPs to adjust their assumptions in response to varied social and economic backgrounds and expectations of patients, and to apply this understanding in clinical care across settings.

The study has several potential limitations. First, although the panel included healthcare professionals with relevant experience, physicians constituted the majority of the panel. Consequently, the perspectives of other stakeholders (i.e., nurses and medical clerks) may have been underrepresented, and the identified competency list may reflect a predominantly physician‐centered view. This limitation may be particularly relevant for competencies related to care coordination, information sharing, and role adjustment across locations, which frequently involve collaboration among multiple healthcare professionals and non‐clinical personnel. Further studies should seek to incorporate a broader range of stakeholders to ascertain whether additional competencies or alternative perspectives emerge in relation to dual‐location resident care. Second, patients and dual‐location residents did not participate in this study. Given the importance of patient involvement and partnerships in research [47], future attempts should receive patient feedback. Third, participation was limited to study participants with prior experience in providing care for dual‐location residents or in supervising trainees involved in such care in resort and non‐metropolitan areas of Nagano Prefecture, Japan. Consequently, the identified competencies may reflect characteristics of dual‐location resident care in such settings, where seasonal migration and second‐home use are relatively common. Although we did not focus only on aspects of patient care specific to the Nagano Prefecture situation during Delphi rounds, it is unclear whether our findings can be generalized to other settings. Fourth, the present study did not explicitly define patterns or motivations of dual‐location living during the Delphi process. As these characteristics may influence healthcare needs and the competencies required of GPs/FPs, future studies should examine whether the applicability of the proposed model in this study varies across different forms of dual‐location living.

Allowing for these limitations, the present study also has several strengths. A key strength is its focus on an emerging yet under‐explored area of clinical practice, namely the care of dual‐location residents, whose numbers are expected to increase in Japan and potentially globally. Previous studies on seasonal migrants and snowbirds have largely focused on healthcare utilization patterns and continuity of care across locations. While these studies have identified important challenges associated with geographically mobile lifestyles, they have not translated these challenges into a competency framework for frontline physicians. The present study addresses this gap by proposing the ROAM model, which summarizes competencies considered important by the Delphi panel for GPs/FPs involved in dual‐location resident care. A second strength of this study is its use of the Delphi method, a widely recognized approach for establishing content validity through expert consensus. Additionally, the high response rate in our study demonstrates strong panel engagement and continuity throughout the Delphi process. Furthermore, the ROAM model provides a useful conceptual lens through which dual‐location resident care can be considered. Although we use the acronym ROAM for ease of communication, the model should be interpreted as a consensus‐based framework derived from a Delphi panel composed predominantly of physicians with experiences in dual‐location resident care in resort and non‐metropolitan settings in Nagano Prefecture, rather than a definitive or universally applicable competency standard. Its transferability to other clinical settings remains to be established through future research.

Our ROAM model has some implications for clinical practice, medical education, and research. As dual‐location living is expected to become increasingly common in Japan and potentially in other settings, the competencies identified in the present study are likely to obtain greater relevance over time. For clinical practice, the model offers a structured framework to guide GPs/FPs, particularly trainees, in managing dual‐location resident care. Medical educators working in areas where dual habitation is relatively common, such as the numerous resort and second‐home areas in Nagano Prefecture, may find this model informative for curricular development aimed at developing competencies for dual‐location resident care. Additionally, the model will be helpful for future research that examines the relationship between the competencies listed in the ROAM model and patient outcomes. Thus, as dual‐location living continues to evolve, the model will likely play an increasingly important role, and further research into its clinical and educational feasibility and influence on patient outcomes is warranted.

## 5 Conclusions

This study developed a consensus‐based competency framework for GPs/FPs involved in the care of dual‐location residents in Japan. The framework consists of 10 competency items organized into four domains and is referred to as the ROAM model. While the model was derived from a Delphi panel composed predominantly of physicians with experience of caring for dual‐location residents in resort and non‐metropolitan areas in Nagano Prefecture, it offers an initial framework for understanding the competencies that may be required in this emerging area of practice. As dual‐location living is expected to become more prevalent, further research is warranted to examine the applicability of the ROAM model across different settings, as well as its potential implications for clinical practice, medical education, and patient outcomes.

## Author Contributions

**Hirohisa Fujikawa:** conceptualization, investigation, funding acquisition, writing – original draft, methodology, validation, formal analysis, project administration, data curation. **Junki Mizumoto:** conceptualization, validation, writing – review and editing, formal analysis, supervision, methodology. **Hirotake Mori:** conceptualization, methodology, validation, writing – review and editing, formal analysis, supervision. **Yuji Nishizaki:** conceptualization, methodology, validation, writing – review and editing, formal analysis, supervision. **Yuichiro Yano:** conceptualization, methodology, validation, writing – review and editing, formal analysis, supervision. **Toshio Naito:** conceptualization, methodology, validation, writing – review and editing, formal analysis, supervision.

## Funding

This work was supported by the Japan Society for the Promotion of Science, Japan (JP24K20148).

## Ethics Statement

Ethical clearance was obtained from the Ethics Committee of Juntendo University Faculty of Medicine (No. E25‐0037).

## Consent

All study participants provided informed consent in the online questionnaire before participating in the study.

## Conflicts of Interest

The authors declare no conflicts of interest.

## Supporting information


**Data S1:** Questionnaire items in Round 1 (in Japanese).
**Data S2:** Questionnaire items in Round 2 (in Japanese).
**Data S3:** The final competency list in Japanese.

## Data Availability

The data that support the findings of this study are available on request from the corresponding author. The data are not publicly available due to privacy or ethical restrictions.

## References

[1] L. Kou , H. Xu , and M. P. Kwan , “Seasonal Mobility and Well‐Being of Older People: The Case of ‘Snowbirds’ to Sanya, China,” Health & Place 54 (2018): 155–163. 30269019 10.1016/j.healthplace.2018.08.008

[2] M. M. Jeffery , J. Wolfson , S. K. Meier , B. E. Dowd , J. M. Abraham , and R. L. Kane , “Health Care Service Use Among Elderly Seasonal Migrators,” Population Health Management 21, no. 5 (2018): 415–421. 29393807 10.1089/pop.2017.0155

[3] K. E. McHugh and R. C. Mings , “Seasonal Migration and Health Care,” Journal of Aging and Health 6, no. 1 (1994): 111–132. 10131552 10.1177/089826439400600107

[4] J. Pickering , V. A. Crooks , J. Snyder , and T. Milner , “Opportunities and Challenges in Providing Health Care for International Retirement Migrants: A Qualitative Case Study of Canadians Travelling to Yuma, Arizona,” Tropical Diseases, Travel Medicine and Vaccines 6 (2020): 9. 32518667 10.1186/s40794-020-00110-6 PMC7268515

[5] S. Z. Shariff , J. M. Paterson , S. N. Dixon , A. X. Garg , and K. K. Clemens , “Prevalence of Winter Migration to Warmer Destinations Among Ontarians (‘Snowbirds’) and Patterns of Their Use of Health Care Services: A Population‐Based Analysis,” CMAJ Open 9, no. 2 (2021): E491–E499. 10.9778/cmajo.20200270 PMC8157986 33990363

[6] S. Kodai , “Reality of ‘Dual Habitation’ in Japan and Its Relationship to Regional Revitalization: Case Study of the City of Minamiboso and Surrounding Area in Chiba Prefecture,” Geographical Review of Japan, Series A 94, no. 5 (2021): 348–363.

[7] M. Sato , “Living in Two Places as Related Population: Expansion of Urban Functions and Realization of Quality of Life in Japan,” in The Palgrave Handbook of Global Social Change (Palgrave Macmillan, 2023).

[8] S. Klien , “Post‐Pandemic Developments in Lifestyle Migration in Japan: From Back‐to‐the‐Land to Urbanrural?,” Journal of Rural Studies 114 (2025): 103505.

[9] M. Kotsubo and T. Nakaya , “Trends in Internal Migration in Japan, 2012–2020: The Impact of the COVID‐19 Pandemic,” Population, Space and Place (2023): e2634. 10.1002/psp.2634 PMC9878244 36718313

[10] Ministry of Land, Infrastructure, Transport, and Tourism , “Guidelines for the Operation of the Act on Development of Infrastructures for Wide‐Area Revitalization (to Promote Dual Residence, etc.),” (accessed February 19, 2026) (2024), https://www.mlit.go.jp/kokudoseisaku/content/001769193.pdf.

[11] Nagano Prefecture , “Promotion of Dual Habitation,” (accessed February 19, 2026) (2026), https://www.pref.nagano.lg.jp/shinko/iju/2chiiki.html.

[12] Yamanashi Prefecture , “Dual Habitation: Relocation and Settlement,” (accessed February 19, 2026) (2026), https://www.pref.yamanashi.jp/try_yamanashi/nikyotenkyoju/index.html.

[13] National Public‐Private Partnership Platform for Promoting Dual Habitation , “National Public‐Private Partnership Platform for Promoting Dual Habitation,” (2026), https://www.2chiiki.org/.

[14] H. Fujikawa , T. Ando , A. Endo , et al., “Competencies Related to Generalism for Japanese Medical Undergraduates: Essential Skills for Comprehensive Care,” Medical Teacher 46, no. sup1 (2024): S21–S30. 39545503 10.1080/0142159X.2024.2385133

[15] H. Fujikawa , H. Tamune , Y. Nishizaki , et al., “Undergraduate General Medicine Education in Japan: A Nationwide Cross‐Sectional Survey of Medical Trainees' Perspectives,” Journal of General and Family Medicine 26, no. 2 (2025): 148–156. 40061388 10.1002/jgf2.752 PMC11890055

[16] K. C. Stange , “The Problem of Fragmentation and the Need for Integrative Solutions,” Annals of Family Medicine 7, no. 2 (2009): 100–103. 19273863 10.1370/afm.971 PMC2653966

[17] S. H. Cheng , C. C. Chen , and Y. F. Hou , “A Longitudinal Examination of Continuity of Care and Avoidable Hospitalization: Evidence From a Universal Coverage Health Care System,” Archives of Internal Medicine 170, no. 18 (2010): 1671–1677. 20937927 10.1001/archinternmed.2010.340

[18] L. M. Kern , J. B. Ringel , M. Rajan , et al., “Ambulatory Care Fragmentation, Emergency Department Visits, and Race: A Nationwide Cohort Study in the US,” Journal of General Internal Medicine 38, no. 4 (2023): 873–880. 36417133 10.1007/s11606-022-07888-5 PMC10039160

[19] M. J. Romano , J. B. Segal , and C. E. Pollack , “The Association Between Continuity of Care and the Overuse of Medical Procedures,” JAMA Internal Medicine 175, no. 7 (2015): 1148–1154. 25984883 10.1001/jamainternmed.2015.1340 PMC5577558

[20] N. R. Gowda , A. Kumar , and S. K. Arya , “The Information Imperative: To Study the Impact of Informational Discontinuity on Clinical Decision Making Among Doctors,” BMC Medical Informatics and Decision Making 20, no. 1 (2020): 175. 32723340 10.1186/s12911-020-01190-2 PMC7388506

[21] W. P. Newton , M. Magill , W. Barr , G. Hoekzema , S. Karuppiah , and K. Stutzman , “Implementing Competency Based ABFM Board Eligibility,” Journal of American Board of Family Medicine 36, no. 4 (2023): 703–707.

[22] Royal Australian College of General Practitioners , “The Clinical Competencies for the CCE,” (2024), https://www.racgp.org.au/getattachment/88f6c871‐21ea‐4090‐9c64‐eb5db6493e76/The‐Clinical‐Competencies‐for‐the‐CCE.aspx.

[23] Japan Primary Care Association , “New Certification System for Japan Primary Care Association‐Certified Family Physicians,” (accessed April 11, 2026) (2026), https://shin‐kateiiryo.primary‐care.or.jp/aims.

[24] Japanese Medical Specialty Board , “Standards, Rules, and Regulations Related to General Medicine,” (accessed April 11, 2026) (2026), https://jbgm.org/menu/%e5%9f%ba%e6%ba%96%e3%83%bb%e7%b4%b0%e5%89%87%e7%ad%89/#sec_02714545191613022025.

[25] A. Kubo and K. Yamamoto , “Grasping the Spatial Transition of Kyu‐ and Shin‐Karuizawa Areas Using the Analysis of the Location of Summer Houses, and Tourist and Recreational Facilities,” Journal of the Japanese Institute of Landscape Architecture 87, no. 5 (2024): 537–542.

[26] H. Koyama and T. Otsuki , “A Study on Gradual Formation of Living Base Places on the Moving Process to the Villas Area in Hara Village, Nagano Prefecture,” Journal of Architecture and Planning (Transactions of AIJ) 87, no. 802 (2022): 2319–2328.

[27] B. Allemang , K. Sitter , and G. Dimitropoulos , “Pragmatism as a Paradigm for Patient‐Oriented Research,” Health Expectations 25, no. 1 (2022): 38–47. 34748689 10.1111/hex.13384 PMC8849373

[28] F. Hasson , S. Keeney , and H. McKenna , “Research Guidelines for the Delphi Survey Technique,” Journal of Advanced Nursing 32, no. 4 (2000): 1008–1015. 11095242

[29] S. Humphrey‐Murto , L. Varpio , C. Gonsalves , and T. J. Wood , “Using Consensus Group Methods Such as Delphi and Nominal Group in Medical Education Research,” Medical Teacher 39, no. 1 (2017): 14–19. 27841062 10.1080/0142159X.2017.1245856

[30] F. Hasson , S. Keeney , and H. McKenna , “Revisiting the Delphi Technique—Research Thinking and Practice: A Discussion Paper,” International Journal of Nursing Studies 168 (2025): 105119. 40383005 10.1016/j.ijnurstu.2025.105119

[31] S. Junger , S. A. Payne , J. Brine , L. Radbruch , and S. G. Brearley , “Guidance on Conducting and REporting DElphi Studies (CREDES) in Palliative Care: Recommendations Based on a Methodological Systematic Review,” Palliative Medicine 31, no. 8 (2017): 684–706. 28190381 10.1177/0269216317690685

[32] C. Powell , “The Delphi Technique: Myths and Realities,” Journal of Advanced Nursing 41, no. 4 (2003): 376–382. 12581103 10.1046/j.1365-2648.2003.02537.x

[33] P. Dielissen , P. Verdonk , B. Bottema , A. Kramer , and T. Lagro‐Janssen , “Expert Consensus on Gender Criteria for Assessment in Medical Communication Education,” Patient Education and Counseling 88, no. 2 (2012): 189–195. 22365589 10.1016/j.pec.2012.01.013

[34] J. Mizumoto , T. Mitsuyama , S. Kondo , M. Izumiya , S. Horita , and M. Eto , “Defining the Observable Processes of Patient Care Related to Social Determinants of Health,” Medical Education 57, no. 1 (2023): 57–65. 35953461 10.1111/medu.14915

[35] H. A. von der Gracht , “Consensus Measurement in Delphi Studies,” Technological Forecasting and Social Change 79, no. 8 (2012): 1525–1536.

[36] A. Homberg , “Conducting Delphi Surveys in Medical Education Research,” GMS Journal for Medical Education 43, no. 1 (2026): Doc6. 41657931 10.3205/zma001800 PMC12875060

[37] R. Hordijk , K. Hendrickx , K. Lanting , A. MacFarlane , M. Muntinga , and J. Suurmond , “Defining a Framework for Medical Teachers' Competencies to Teach Ethnic and Cultural Diversity: Results of a European Delphi Study,” Medical Teacher 41, no. 1 (2019): 68–74. 29490534 10.1080/0142159X.2018.1439160

[38] A. L. Hixon , “Contextualized Care Not a New Concept in Family Practice,” Academic Medicine 77, no. 10 (2002): 945. 12377665 10.1097/00001888-200210000-00002

[39] R. Ohta , A. Ueno , J. Kitayuguchi , Y. Moriwaki , J. Otani , and C. Sano , “Comprehensive Care Through Family Medicine: Improving the Sustainability of Aging Societies,” Geriatrics 6, no. 2 (2021): 59. 34199871 10.3390/geriatrics6020059 PMC8293036

[40] T. Ando , T. Sasaki , Y. Abe , et al., “Measurement of Polydoctoring as a Crucial Component of Fragmentation of Care Among Patients With Multimorbidity: Cross‐Sectional Study in Japan,” Journal of General and Family Medicine 24, no. 6 (2023): 343–349. 38025930 10.1002/jgf2.651 PMC10646296

[41] A. Prior , C. H. Vestergaard , P. Vedsted , et al., “Healthcare Fragmentation, Multimorbidity, Potentially Inappropriate Medication, and Mortality: A Danish Nationwide Cohort Study,” BMC Medicine 21, no. 1 (2023): 305. 37580711 10.1186/s12916-023-03021-3 PMC10426166

[42] L. M. Kern , M. M. Safford , M. J. Slavin , et al., “Patients' and Providers' Views on Causes and Consequences of Healthcare Fragmentation in the Ambulatory Setting: A Qualitative Study,” Journal of General Internal Medicine 34, no. 6 (2019): 899–907. 30783883 10.1007/s11606-019-04859-1 PMC6544669

[43] H. Fujikawa , T. Aoki , T. Ando , and J. Haruta , “Family Physicians Have Greater Ambiguity Tolerance in the Clinical Context: A Nationwide Cross‐Sectional Study,” Journal of General and Family Medicine 26, no. 2 (2025): 128–134. 40061390 10.1002/jgf2.747 PMC11890053

[44] R. Kumar , S. Bhattacharya , N. Sharma , and A. Thiyagarajan , “Cultural Competence in Family Practice and Primary Care Setting,” Journal of Family Medicine and Primary Care 8, no. 1 (2019): 1–4. 10.4103/jfmpc.jfmpc_393_18 PMC6396634 30911472

[45] A. G. Mainous , Z. Xie , S. Yadav , M. Williams , A. V. Blue , and Y. R. Hong , “Physician Cultural Competency Training and Impact on Behavior: Evidence From the 2016 National Ambulatory Medical Care Survey,” Family Medicine 52, no. 8 (2020): 562–569. 32931004 10.22454/FamMed.2020.163135

[46] K. Hunter and B. Thomson , “A Scoping Review of Social Determinants of Health Curricula in Post‐Graduate Medical Education,” Canadian Medical Education Journal 10, no. 3 (2019): e61–e71. 31388378 PMC6681926

[47] C. Jinks , P. Carter , C. Rhodes , et al., “Patient and Public Involvement in Primary Care Research—An Example of Ensuring Its Sustainability,” Research Involvement and Engagement 2 (2016): 1. 29062502 10.1186/s40900-016-0015-1 PMC5611572

