# Supplementary material for: Developing a Competency List for General Practitioners/Family Physicians Providing Care for Dual‐Location Residents: Expert Consensus via the Delphi Technique
Source: J Gen Fam Med. 2026 Jul 7;27(4):e70149. doi: 10.1002/jgf2.70149 (PMC13341965; doi:10.1002/jgf2.70149)
Supplement: Supplementary file 1 — Data S1: Questionnaire items in Round 1 (in Japanese). Data S2: Questionnaire items in Round 2 (in Japanese). Data S3: The final competency list in Japanese. [file JGF2-27-e70149-s001.pdf]

Supplementary Item S1. Questionnaire items in Round 1 (in Japanese)

本研究の目的は、
「二拠点生活者診療に特に必要な総合診療医の能力を解明すること」
です。

近年、コロナ禍を経て、都市部と地方など２つの場所に生活拠点を持ち、行き来しながら暮らす人（二拠点生活者）が増えており、総合診療医が二拠点生活者の診療に関わる場面も増えています。そこで皆さんがこれまでに経験した、二拠点生活者の診療や支援の場面を思い浮かべながら、以下の問いにお答えください。

なお今回は、

- 総合診療外来
- もう一方の拠点にあるかかりつけ医療機関から紹介状を持参した定期患者診療だけでなく、当日予約外で急遽来院した患者の診療も含みます

Q. 二拠点生活者に対して、総合診療医がよりよい医療を提供するために特に大切だと思う能力には、どのようなものがあるでしょうか。自由にお書きください。なお、

- 本調査に正解・不正解はありません。どのようなご意見でも歓迎いたしますので、率直にお書きください。
- 思いつくものを、できるだけ多く挙げてください。答え方は、単語でも文章でも、形式は問いません。
- 「二拠点生活者の診療に特有の能力」だけでなく、「普段の診療でも必要だが、二拠点生活者の診療では特に重要だと思う能力」も含めて挙げてください。

Supplementary Item S2. Questionnaire items in Round 2 (in Japanese)
改めて本研究の目的を記しますと、
「二拠点生活者診療に特に必要な総合診療医の能力を解明すること」
です。

今回の第 2 ラウンドの調査票では、第 1 ラウンドの結果から作成した、項目リストについて、ご回答いただきます。

各項目について、「二拠点生活者診療に特に必要な総合診療医の能力」として重要であるとあなたが思う程度を、5 段階のなかから 1 つお選びください（1 = 全く重要ではない 〜 5 = 非常に重要である）。また、文章が不適切である、あるいは修正が望ましいと思われる場合には、選択肢の下の自由記述欄に意見や修正点をお書きください。

Q1. 患者の二拠点生活の背景、生活パターン、家族・支援体制を把握できる
1 = 全く重要ではない
2 = あまり重要ではない
3 = どちらでもない
4 = 少し重要である
5 = 非常に重要である
本項目への意見や、文章についての修正案があればご記入ください。

Q2. 地域差（気候や文化など）が健康に与える影響を考慮できる
1 = 全く重要ではない
2 = あまり重要ではない
3 = どちらでもない
4 = 少し重要である
5 = 非常に重要である
本項目への意見や、文章についての修正案があればご記入ください。

Q3. 患者および他拠点の医療機関から必要な医療情報を収集できる
1 = 全く重要ではない
2 = あまり重要ではない
3 = どちらでもない
4 = 少し重要である
5 = 非常に重要である
本項目への意見や、文章についての修正案があればご記入ください。

Q4. 他拠点の医療機関と適切に情報共有ができる

1 = 全く重要ではない
2 = あまり重要ではない
3 = どちらでもない
4 = 少し重要である
5 = 非常に重要である
本項目への意見や、文章についての修正案があればご記入ください。

Q5. 二拠点間での診療の役割分担や関与の程度を状況に応じて調整できる

1 = 全く重要ではない
2 = あまり重要ではない
3 = どちらでもない
4 = 少し重要である
5 = 非常に重要である
本項目への意見や、文章についての修正案があればご記入ください。

Q6. 情報が不十分な状況でも、必要な対応の優先順位を判断できる

1 = 全く重要ではない
2 = あまり重要ではない
3 = どちらでもない
4 = 少し重要である
5 = 非常に重要である
本項目への意見や、文章についての修正案があればご記入ください。

Q7. 二拠点生活に伴う受診行動や医療機関受診のあり方を患者と調整できる

1 = 全く重要ではない
2 = あまり重要ではない
3 = どちらでもない
4 = 少し重要である
5 = 非常に重要である
本項目への意見や、文章についての修正案があればご記入ください。

Q8. 将来の療養方針や生活について患者と話し合うことができる

1 = 全く重要ではない
2 = あまり重要ではない

3 = どちらでもない
4 = 少し重要である
5 = 非常に重要である
本項目への意見や、文章についての修正案があればご記入ください。

Q9. 二拠点生活に伴う社会的・文化的背景や医療への期待の違いを踏まえ、自身の認識を調整できる

1 = 全く重要ではない
2 = あまり重要ではない
3 = どちらでもない
4 = 少し重要である
5 = 非常に重要である
本項目への意見や、文章についての修正案があればご記入ください。

Q10. 二拠点生活に伴う社会的・文化的背景や医療への期待の違いに配慮した対応ができる

1 = 全く重要ではない
2 = あまり重要ではない
3 = どちらでもない
4 = 少し重要である
5 = 非常に重要である
本項目への意見や、文章についての修正案があればご記入ください。

Supplementary Item S3. The final competency list in Japanese

1. 患者の二拠点生活の背景、生活パターン、家族・支援体制を把握できる
2. 二拠点の地域差が健康や生活に与える影響を考慮できる
3. 患者および他拠点の医療機関から必要な医療情報を収集できる
4. 他拠点の医療機関と適切に情報共有ができる
5. 二拠点間での診療の役割分担や関与の程度を状況に応じて調整できる
6. 情報が不十分な状況でも、必要な対応の優先順位を判断できる
7. 二拠点生活に伴う受診行動や医療機関受診のあり方を患者と調整できる
8. 将来の療養方針や生活について患者と話し合うことができる
9. 二拠点生活に伴う社会的・文化的背景や医療への期待の違いを踏まえ、自身の認識を調整できる
10. 二拠点生活に伴う社会的・文化的背景や医療への期待の違いに配慮した対応ができる

※ こちらのコンピテンシー・リストを使用する際は、責任著者にメールで許諾を得たうえで、本論文の引用をお願いいたします。
